# Increased BMI and Blood Lipids Are Associated With a Hypercoagulable State in the Moli-sani Cohort

**DOI:** 10.3389/fcvm.2022.897733

**Published:** 2022-06-16

**Authors:** Romy de Laat-Kremers, Augusto Di Castelnuovo, Lisa van der Vorm, Simona Costanzo, Marisa Ninivaggi, Chiara Cerletti, Dana Huskens, Amalia De Curtis, Alessandro Gialluisi, Cuicui Bai, Giovanni de Gaetano, Dongmei Yin, Maria Benedetta Donati, Bas de Laat, Licia Iacoviello, The Moli-sani Investigators

**Affiliations:** ^1^Department of Data Analysis and Artificial Intelligence, Synapse Research Institute, Maastricht, Netherlands; ^2^Mediterranea Cardiocentro, Naples, Italy; ^3^Department of Functional Coagulation, Synapse Research Institute, Maastricht, Netherlands; ^4^Department of Epidemiology and Prevention, IRCCS Neuromed, Pozzilli, Italy; ^5^Department of Platelet Pathophysiology, Synapse Research Institute, Maastricht, Netherlands; ^6^Research Center in Epidemiology and Preventive Medicine (EPIMED), Department of Medicine and Surgery, University of Insubria, Varese, Italy; ^7^Department of Protein Engineering, Synapse Research Institute, Maastricht, Netherlands

**Keywords:** lipids, BMI, Moli-sani, thrombin, thrombin generation

## Abstract

The coagulation system can be assessed by the thrombin generation (TG) assay, and increased TG peak height, endogenous thrombin potential (ETP), and velocity index are associated with an increased risk of thrombosis. Obesity had been reported to increase TG and is associated with dyslipidemia, which also predisposes to atherosclerotic cardiovascular disease (CVD). However, the effect of the blood lipid profile on TG has not been studied extensively. To gain more insight into the associations of TG, body mass index (BMI) and lipid profile, we studied TG in relation to these parameters in a large Italian population cohort, the Moli-sani study (*N* = 22,546; age ≥ 35 years; 48% men). TG was measured in plasma samples collected at the enrollment of subjects in the Moli-sani study. TG was triggered with 1 or 5 pM tissue factor, and TG parameters lag time, peak, ETP, time-to-peak (TTP) and velocity index (VI). Additionally, thrombomodulin was added to assess the function of the activated protein C system during TG. In both women and men, overweight (BMI 25–30 kg/m^2^) and obesity (BMI > 30 kg/m^2^) were significantly associated with higher ETP, peak and VI (all *p* < 0.001). High total cholesterol, triglycerides and LDL-cholesterol levels were significantly associated with increased ETP and peak (all *p* < 0.001). Linear regression analysis revealed that the ETP is positively associated with both plasma LDL and HDL cholesterol levels, whereas the velocity index is positively associated with HDL cholesterol. Additionally, ETP, peak and VI were significantly associated with the plasma triglycerides content. In conclusion, our study shows significant associations of high BMI and blood lipid levels with increased TG parameters, and this hypercoagulability may partly explain the increased risk of CVD in individuals with obesity and/or dyslipidemia.

## Introduction

Cardiovascular disease (CVD) remains among the most important chronic diseases, contributing to more than 17 million deaths per year globally (1). A major cause of CVD is atherosclerosis, characterized by the formation of a plaque or lesion in artery walls (2). One of the key risk factors for atherosclerotic CVD events is the blood lipid profile: low-density lipoprotein cholesterol (LDL-C) and high-density lipoprotein cholesterol (HDL-C) have opposite effects on CVD risk, consistent with the role of LDL and HDL particles in promoting and protecting against atherosclerosis, respectively (3–5). Overweight and obesity [defined as a body mass index (BMI) of more than 25 or more than 30 kg/m^2^, respectively (6)] are key risk factors for dyslipidemia (7) and independently associated with an increased risk for arterial and venous thrombotic CVD (8, 9). On the other hand, abnormalities in coagulation and hemostasis represent a well-known link in the relationship between increased BMI and thrombotic risk.

Interestingly, in many individuals with obesity, changes in the hemostatic and fibrinolytic activity are observed that lead to hypercoagulability. For instance, obesity has been associated with elevated levels of tissue factor (TF), coagulation factor (F)VII and FVIII, von Willebrand factor and fibrinogen (10, 11). Furthermore, obese patients present a hypofibrinolytic state owing to higher levels of the plasminogen activator inhibitor 1 (12). Overall, the resulting pro-thrombotic state may contribute to the development of CVD and venous thromboembolism (VTE) in patients with obesity. A promising tool to assess an individuals' coagulation potential is the measurement of the *in vitro* thrombin generation (TG) profile. TG is a global coagulation assay that describes the potential of a blood (13) or plasma (14) sample to generate thrombin and promote coagulation. It allows the quantification of the combined effects of changes in pro- and anticoagulant factors, thus predicting if there is an increased risk of bleeding or thrombosis (15, 16). Indeed, increased TG was found to be associated with first- and recurrent VTE (17, 18), and with myocardial infarction (19).

Obesity has been reported to increase TG in case-control (20) and small cohort studies (21), and weight loss has been shown to reduce TG (22). However, the effect of the blood lipid profile on TG has not been studied extensively. Mechanistically, LDL-C has been reported to support the assembly of the pro-thrombinase complex, and thereby enables the generation of thrombin (23, 24). Moreover, statins have been reported to lower TG, but this antithrombotic effect might be not related to their lipid-lowering properties, at least in part (25). A study by Kim et al. (26) in healthy subjects (*n* = 448) reported significant correlations between several TG parameters and levels of total cholesterol, LDL-C, HDL-C and triglycerides. However, this study did not include clinical data nor medical history, and no data on well-known risk factors for hypercoagulable status-such as obesity–was collected. Therefore, a large cohort study is needed to gain further insights into the associations between TG, lipids and BMI. The Moli-sani cohort consists of 24,325 subjects from the general population of whom detailed information on diet, lifestyle and information on morbidity and mortality was collected. We measured TG profiles in plasma samples from the participants of the Moli-sani cohort, with the aim to investigate the association of BMI and blood lipids content with TG.

## Methods

### Study Population

**Figure 1:**
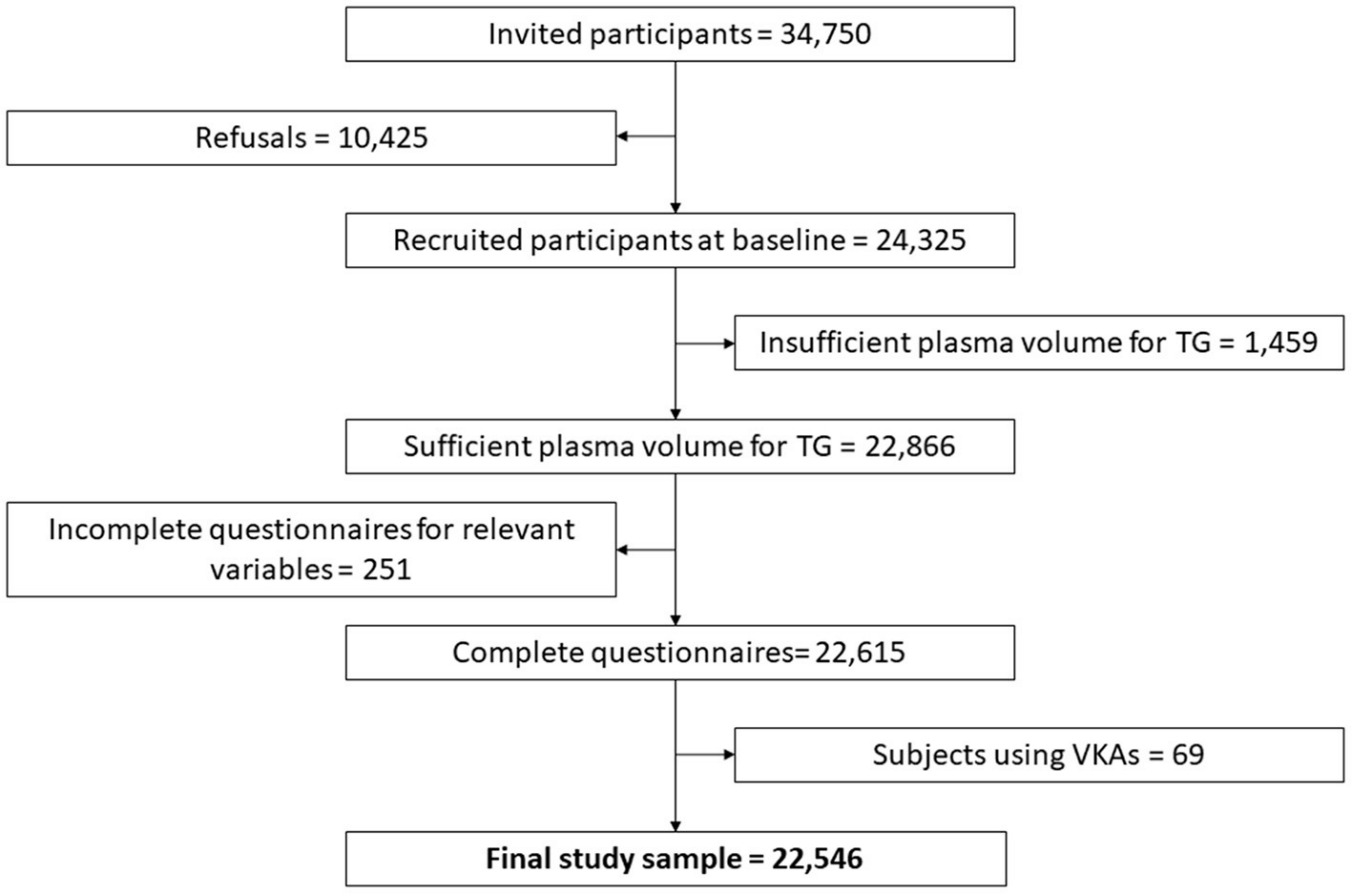
Flowchart of selection of the studied population among Moli-sani participants. The groups of eliminated participants (of the 24 325 recruited at baseline) overlap and the scheme shows sequential exclusion criteria. Therefore, the patients in each eliminated group do not fulfill the previous elimination criteria, but it is possible that patients on VKAs were previously eliminated because of e.g., insufficient plasma volume for thrombin generation.

The Moli-sani cohort was randomly recruited in the Molise region (Italy) from city hall registries, as previously described (27, 28). The Moli-sani study complies with the Declaration of Helsinki and was approved by the ethics committee of the Catholic University of Rome, Italy. In total, 24, 325 subjects were enrolled between March 2005 and April 2010. All participants provided written informed consent. For the current study, TG was measured in a total of 22,866 subjects, due to a lack of sufficient plasma volume for the remaining participants (Figure 1). Additionally, subjects with incomplete baseline questionnaires were excluded, as well as subjects using anticoagulants, leaving 22,546 observations for statistical analysis. At the time of enrollment in the study, none of the subjects received direct oral anticoagulants.

### Blood Collection, Plasma Preparation and Storage

Venous blood samples were previously obtained by venipuncture between 07:00 am and 09:00 am from participants who had fasted overnight and had refrained from smoking for at least 6 h (27). Citrated plasma samples for this study were stored in straws containing the sample code and barcode in liquid nitrogen in a dedicated biobank (http://www.neuromed.it/biobanking-centre/) for 8.2 years (IQR: 7.2 to 9.2 years). Platelet poor plasma was prepared by centrifugating twice at 2,821 g for 10 min. The samples were express-shipped in 3 shipments on dry ice to Synapse Research Institute, Maastricht, the Netherlands on 27-10-2016, 08-05-2017 and 23-06-2017, where they were immediately stored at−80°C. Levels of labile coagulation factors (FV and FVIII) were determined in a subset of 144 randomly chosen samples from the first shipment to confirm plasma sample quality. FV and FVIII levels were measured on the STA-R device using STA deficient FV and STA deficient FVIII reagents, according to the manufacturer's specifications (Diagnostica Stago, Asnières, France). All coagulation factor levels measured were in line with the reference ranges, established by the manufacturer.

### Thrombin Generation Measurements

TG was determined in platelet-poor plasma (PPP) using the Calibrated Automated Thrombinography (CAT) method (29). Due to the number of samples in the cohort, every condition was measured in a single well, for each sample. To standardize the measurement procedure as much as possible, commercial reagents from one batch were used to measure TG for all samples. Commercial calibrator, PPP Reagent Low, PPP Reagent [with and without thrombomodulin (TM)] and FluCa kits were purchased from Diagnostica Stago (Asnières, France). All reagents were stored at 4°C, according to the manufacturer's recommendation. Analyses were performed in 96-well round bottom microplates (Immulon 2 HB plate, Fisher Scientific, Roskilde, Denmark). Each well contained 20 μL PPP Reagent Low, PPP Reagent or calibrator and 80 μL plasma. The samples were preincubated for 10 min and analysis was performed at 37°C in a fluorescence plate reader (Fluoroskan Ascent, Diagnostica Stago, Asnières, France). TG curves and calibration curves were measured immediately after the automatic dispensing of 20 μL FluCa. The TG curve was monitored for 50 min, or shorter if all curves reached the baseline level earlier, to ensure that the TG curves could be calculated in a reliable way. TG curves were measured and analyzed by the Thrombinoscope software (version 5.0), as specified by the manufacturer (Diagnostica Stago, Asnières, France). TG parameters lagtime (time until the first trace of thrombin is formed), peak height (maximum amount of active thrombin present), time-to-peak (TTP, time until the maximum amount of active thrombin is present), area-under-the-curve (endogenous thrombin potential, ETP), and maximum slope of thrombin formation (velocity index, VI) were quantified automatically by the software.

### Determination of CVD Risk Factors at Baseline

Blood glucose, serum lipids and C-reactive protein (CRP) were measured within 3 h from blood sampling by enzymatic reaction methods using an automatic analyzer (ILab 350, Milano, Italy). LDL-C level was calculated according to the Friedewald formula (30). Height and weight were measured at baseline, and body mass index (BMI) was calculated as kg/m^2^. For specific analyses, BMI was grouped into three categories as normal (<25 kg/m^2^), overweight (25–30 kg/m^2^) or obese (>30 kg/m^2^), according to WHO guidelines (6). A status of “current smoker” was defined as smoking 1 or more cigarettes per day on a regular basis. Waist and hip circumferences were measured according to the WHO guidelines (31) and used to calculate the waist-to-hip (WH) ratio.

### Statistical Analyses

The normality of continuous variables was assessed graphically in histograms and normality Q-Q plots (data not shown). Distributions of serum triglycerides, blood glucose, and serum CRP concentrations were skewed and are therefore presented as median and interquartile range (IQR). Values for continuous non-skewed variables are presented as means ± SD. Categorical variables are presented as frequencies. Comparisons of continuous variables between two groups or more than two groups were performed using the Student's *t*-test or Mann-Whitney U test and analysis of variance F test, respectively. Associations between continuous variables were tested using the Spearman's rank correlation coefficient.

The effect of blood lipid levels (total cholesterol, triglycerides, LDL-C, HDL-C) and BMI on TG parameters was evaluated by linear regression analyses. Crude linear regression models stratified by gender were generated with the TG parameters ETP/Peak/LT/TTP/VI as main outcomes and BMI or the different types of lipid parameters (total cholesterol, LDL-C, HDL-C, triglycerides) as exposures. To build the multivariate models, the following strategy was applied: (1) simple univariate regression (UVR) analysis was used to identify independent/predictor variables associated with the dependent/outcome variable (ETP, peak, VI) at the level *P* < 0.05; (2) all the variables identified in the univariate analysis were inserted in a multiple regression (MR) analysis. For all models, the normality of residuals and homoscedasticity were evaluated by plotting standardized residuals against the predicted values. In case of violation of one or both of these conditions, the variable was not included in the model. The presence of collinearity among independent variables was tested by the variance inflation factor (VIF). A VIF of ≥10 indicates that the variable under consideration is almost a perfect linear combination of the independent variables already in the equation, in which case the variable is not added to the regression equation.

Further associations between TG parameters and general laboratory results and patient characteristics were performed by dividing the TG parameters into quartiles and performing ANOVA analysis on the groups to detect significant differences between high and low parameter values.

A two-sided *P*-value <0.05 was considered statistically significant for all analyses. IBM Statistical Package for Social Sciences (SPSS) version 25 software was used for all statistical analyses (SPSS Incorporated, Chicago, USA). Figures were prepared using GraphPad Prism version 5.00 (GraphPad Software, San Diego, USA).

## Results

### Moli-sani Population

We first determined baseline general characteristics for the subsection of the Moli-sani cohort in which we studied TG (Table 1). Our study population consisted of slightly more women (*n* = 11,766; 52.2%) than men (*n* = 10,780; 47.8%). On average, male subjects were older, more frequently smoking and had a slightly higher BMI than female subjects. Men had lower total cholesterol, LDL-C as well as HDL-C levels than women, although these differences were small. In contrast, male subjects had substantially higher triglyceride levels than female subjects. Blood glucose was higher in men, whereas CRP levels were slightly higher in women.

**Table 1:** General characteristics of the Moli-sani cohort, stratified by sex.

**Variable**	**Women (*n* = 11,766)**	**Men (*n* = 10,780)**	***P*-value**
Age, *y*	55.1 ± 11.7	56.1 ± 11.9	<0.001
Current smoker, %	20.4	26.1	<0.001
BMI, *kg/m^2^*	27.9 ± 5.3	28.2 ± 4.0	<0.001
Waist:hip ratio	0.89 ± 0.09	0.95 ± 0.06	<0.001
Total cholesterol, *mg/dL*	216.5 ± 41.6	210.1 ± 41.8	<0.001
LDL-C, *mg/dL*	131.4 ± 35.4	129.1 ± 34.9	<0.001
HDL-C, *mg/dL*	62.8 ± 14.7	51.9 ± 12.9	<0.001
Triglycerides, *mg/dL*	97 (63)	124 (88)	<0.001
Blood glucose, *mg/dL*	93 (16)	100 (19)	<0.001
CRP, *mg/L*	1.56 (2.36)	1.47 (2.00)	0.002

*Data are presented as the mean ± SD or median (IQR) for normally distributed and skewed continuous variables, respectively, and as a percentage for categorical variables. Statistical differences between men and women were analyzed using a Student's t-test or Mann-Whitney U test for normally distributed and skewed continuous variables, respectively, and a Pearson's χ^2^ test for categorical variables. BMI, Body Mass Index; LDL-C, low density lipoprotein cholesterol; HDL-C, high density lipoprotein cholesterol; CRP, C-reactive protein*.

TG [as well as the risk for bleeding and thrombosis (32, 33)] is known to be influenced by age (34) and sex (35). Table 2 presents TG parameters measured in platelet poor plasma with the two types of TF concentrations, stratified by sex. For both the PPP reagent low and PPP reagent, women had significantly higher TG ETP, peak and VI, in combination with a shorter lagtime and TTP, compared to men. The inhibition of the ETP by thrombomodulin (TM) was significantly reduced in women (ETP_TM−_ = 1,764 ± 436 nM·min vs. ETP_TM+_ = 1,426 ± 536 nM·min) compared to men (ETP_TM−_ = 1,692 ± 418 nM·min vs. ETP_TM+_ = 1,346 ± 518 nM·min). This difference was slightly larger when considering only women taking oral contraceptives (*n* = 934), with an ETP inhibition of 12.11% (IQR 9%) and peak inhibition of 0.66% (IQR 4%), compared to male subjects (*p* <0.001) (data not shown).

**Table 2:** Thrombin generation (TG) parameters in the Moli-sani cohort, stratified by sex.

**TG parameter**	**Women (*n* = 11,766)**	**Men (*n* = 10,780)**	***P*-value**
**PPP Reagent low**			
ETP, *nM min*	1754.7 ± 419.3	1685.4 ± 400.2	<0.001
Peak, *nM*	370.6 ± 89.9	356.8 ± 86.4	<0.001
Lag time, *min*	3.94 (1)	4 (1)	<0.001
Time-to-peak, *min*	6.00 (1)	6.33 (2)	<0.001
VI, *nM/min*	166.2 ± 64.8	160.4 ± 61.6	<0.001
**PPP Reagent**			
ETP, *nM min*	1766.9 ± 433.5	1698.4 ± 412.3	<0.001
Peak, *nM*	374.8 ± 83.4	355.7 ± 79.8	<0.001
Lag time, *min*	2.67 (1)	2.67 (1)	<0.001
Time-to-peak, *min*	5.00 (1)	5.00 (1)	<0.001
VI, *nM/min*	167.8 ± 57.3	156.9 ± 54.7	<0.001
**PPP Reagent** **+** **Thrombomodulin**			
ETP inhibition, %	12.24 (9)	12.63 (11)	<0.001
Peak inhibition, %	0.84 (4)	1.83 (5)	<0.001

*Data are presented as the mean ± SD or median (IQR) for normally distributed and skewed continuous variables, respectively. PPP, platelet poor plasma; ETP, endogenous thrombin potential; TTP, time to peak; VI, velocity index*.

The relationship of the ETP with age was also explored. Both in PPP reagent low and PPP reagent-induced TG, subjects in the lowest quartile of the ETP values were significantly (*p* <0.001) younger (mean age 54.5 ± 11.1 and 54.4 ± 11.1 years) compared to the individuals in the highest quartile of the ETP values (mean age 57.5 ± 12.7 and 57.4 ± 12.7 years), respectively. Additionally, the inhibition of the ETP by the anticoagulant actions of TM was inversely associated with age: subjects in the lowest quartile of ETP inhibition by TM were significantly older [median age 58 (IQR 21)] than the subjects whose ETP responded more to the inhibitory effect of TM [median age 53 (IQR 16)]. The other TG parameters were not significantly associated with age in the current Moli-sani cohort.

### Obesity Is Associated With Increased TG

In both women and men, overweight (BMI 25–30 kg/m^2^) and obesity (BMI>30 kg/m^2^) were associated with an increased TG, characterized by increased ETP, peak and VI and prolonged lagtime and TTP (Supplementary Table 1). Inhibition of the ETP by the anticoagulant actions of TM was on average lower in obese men compared to men in the other BMI categories, while no significant differences were observed for TM-dependent inhibition in women.

These differences were further confirmed in linear regression models, where higher BMI was significantly associated with higher ETP and VI. The BMI of subjects in the upper quartile of the ETP was on average 8% higher than that of subjects in the lowest ETP quartile (*p* < 0.001). The same trend was observed for VI, where BMI was 4% higher in individuals in the upper quartile of VI distribution, compared to those in the lowest quartile (*p* < 0.001). Finally, a high BMI was associated with a more coagulable state of the blood, as measured by TG.

### The Relation Between TG Parameters and Blood Lipid Levels

Since high BMI is related to altered blood lipid composition, we next studied the relationship between lipid profiles and TG in our study population. Blood lipid levels were divided into two groups according to the National Cholesterol Education Program (NCEP) guidelines (36), see Supplementary Table 2. Older individuals had significantly higher (≥240 mg/dL and ≥190 mg/dL) total cholesterol and LDL-C levels, respectively. Whereas, subjects with high triglyceride levels (≥200 mg/dL) were more frequently men, the groups with high total cholesterol (≥240 mg/dL), high LDL-C (≥190 mg/dL) and high HDL-C (≥60 mg/dL) contained more women. BMI, proportion of smokers, WH-ratio, CRP and glucose levels were all significantly higher in individuals with lipid levels above the cutoff threshold, except for HDL-C ≥60 mg/dL, which contained on average fewer smokers and more subjects with lower BMI, WH-ratio, CRP and glucose levels. Similarly, TG parameters were generally significantly increased in individuals with lipid levels above the cutoff, except for HDL-C, for which most differences in TG parameters between the two lipid level categories were non-significant (except for the time-dependent parameters).

The relation between lipids and TG was further explored in an association analysis, based on quartiles of the TG parameters ETP and peak (Table 3). LDL-C and HDL-C showed different associations with TG: individuals in the lowest quartile for ETP and peak had the lowest plasma LDL-C levels, whereas the HDL-C content did not differ between the quartiles. LDL-C levels were 9% higher in subjects in the highest compared to the lowest quartile of peak TG (*p* < 0.001), and 14% higher in those in the highest compared to the lowest quartile of the ETP (*p* < 0.001).

**Table 3:** Linear regression and quartile analysis of lipid levels as determinants for TG parametersdetermined using PPP reagent.

		**Cholesterol mg/dL)**	**Triglycerides (mg/dL)**	**LDL cholesterol (mg/dL)**	**HDL cholersterol (mg/dL)**
Lag time	β-coef. UVR	0.002***	0.001***	0.003***	−0.005***
	β-coef. MR	COL	0.001***	0.003***	−0.005***
	Q1	203 (52)	97 (67)	121 (43)	58 (20)
	Q2	211 (54)***	107 (73)***	128 (44)***	57 (20)***
	Q3	216 (56)***	115 (79)***	134 (45)***	55 (19)***
	Q4	221 (59)***	127 (90)***	137 (49)***	52 (18)***
ETP	β-coef. UVR	1.97***	0.50***	2.17***	
	β-coef. MR	1.13***	0.51***	0.80**	
	Q1	200 (52)	98 (68)	120 (44)	56 (19)
	Q2	209 (54)***	107 (73)***	126 (43)***	56 (20)
	Q3	215 (53)***	112 (79)***	132 (45)***	56 (19)
	Q4	220 (58)***	117 (82)***	136 (48)***	56 (19)
Peak	β-coef. UVR	0.29***	0.10***	0.27***	0.15***
	β-coef. MR	COL	0.20***	0.22***	0.38***
	Q1	204 (56)	99 (70)	124 (47)	55 (20)
	Q2	208 (53)***	105 (74)**	126 (44)***	56 (19)
	Q3	212 (54)***	111 (74)***	129 (44)***	56 (20)
	Q4	219 (56)***	119 (84)***	134 (47)***	56 (19)
Time-to-peak	β-coef. UVR	0.003***	0.001***	0.004***	−0.006***
	β-coef. MR	COL	0.001***	0.005***	−0.006***
	Q1	202 (50)	101 (70)	119 (42)	57 (20)
	Q2	211 (54)***	107 (74)***	128 (45)***	57 (19)*
	Q3	214 (55)***	111 (77)***	132 (46)***	55 (20)***
	Q4	220 (59)***	120 (88)***	137 (49)***	54 (19)***
Velocity index	β-coef. UVR	0.05***	0.04***		0.06*
	β-coef. MR	0.01	0.06***		0.15**
	Q1	211 (57)	128 (47)	105 (74)	55 (19)
	Q2	210 (55)	128 (45)	104 (73)	56 (20)**
	Q3	209 (54)	127 (45)	108 (75)**	56 (20)*
	Q4	214 (54)***	130 (45)***	117 (82)	56 (19)

Linear regression results are presented as β-coefficients and their associated p-values. Lipid levels were quantified as the median and IQR for the quartiles of each TG parameter. (Bonferroni corrected) p-values were indicated as *p <0.05, **p < 0.01, ***p < 0.001. Sex was coded as ‘'0” for men and ‘'1” for women, hence a negative β-coefficient indicates a decrease in outcome (TG) variable in women compared to men. Smoking was coded as ‘'0” for non-smokers and ‘'1” for current smokers, hence a negative β-coefficient indicates a decrease in outcome (TG) variable in smokers compared to non-smokers. Significant variables shown in the table were included in the final model. β-coef., β-coefficient; ETP, Endogenous thrombin potential; UVR, univariate regression analysis; MR, multiple regression analysis; COL, variable excluded from the model because of collinearity (VIF ≥ 10), Q, quartile.

Finally, linear regression analyses were performed to investigate to what degree lipid levels contribute to the (normally distributed) TG parameters ETP, peak and VI (Table 3). In simple UVR, most lipid parameters were significantly associated with each of the TG parameters. Exceptions were HDL-C levels, which were not significantly associated with TG peak height in PPP reagent low-induced TG and with ETP in PPP reagent-induced TG, and LDL-C which was not associated with the VI in PPP reagent-induced TG. Triglyceride levels significantly contributed to PPP reagent low and PPP reagent-induced TG parameters. Total cholesterol had limited added predictive value for TG parameters (also due to collinearity) and was hence not included in most of the final MR models. ETP and peak were mainly affected by triglycerides and LDL-C, whereas VI was also influenced by HDL-C for both TG conditions. Overall, *R*^2^ values of the final models were low, indicating that only a small proportion of the variance in TG parameters could be explained by lipid levels.

### The Contribution of BMI, Lipids and Related Factors to TG

Many factors are known to influence TG, besides the blood lipid profile. Table 1 summarizes the most important variables that affect TG parameters. To adjust for these variables, multiple linear regression analysis was performed for all TG variables to investigate whether, and to what degree the lipid profile influences TG results, adjusting for other differences related to the lipid profile between the subjects (Table 4).

**Table 4:** Determinants for TG parameters in linear regression analysis.

	**Lag time**		**ETP**		**Peak**		**Time-to-peak**		**Velocity index**	
	**β-coefficient**		**β-coefficient**		**β-coefficient**		**β-coefficient**		**β-coefficient**	
**PPP reagent low**	**UVR**	**MR**	**UVR**	**MR**	**UVR**	**MR**	**UVR**	**MR**	**UVR**	**MR**
Age, y	0.003***	0.001*	−3.51***	−4.93***			−0.005***	−0.005***	0.47***	0.24***
Sex	0.17***	0.092***	−69.4***	−55.5***	−13.9***	−15.0***	0.168***	0.148***	−5.74***	−9.88***
BMI, kg/m2	0.025***	0.008***	14.0***	12.4***	2.55***	1.46***	0.017***	0.013***	2.55***	0.47***
Smoking	0.038***		−14.6***		−3.19***		0.026*			
Waist:hip ratio	1.191***		111**		68.7***	21.3*	0.334*	−0.645***	65.7***	29.9***
Total cholesterol, mg/dL	0.003***	−0.006***	2.02***	COL	0.34***	COL	0.003***	COL	0.10***	COL
LDL-C, mg/dL	0.004***	0.009***	2.24***	1.87***	0.33***	COL	0.005***	0.005***	0.07***	COL
HDL-C, mg/dL	−0.009***	COL	0.45*	1.79***			−0.009***	−0.007***	−0.09**	−0.49***
TGL, mg/dL	0.002***	0.002***	0.49***	0.63***	0.14***	0.08***	0.001***	0.001**	0.08***	
Blood glucose, mg/dL	0.001***	−0.001***	−0.32**	−0.93***	0.16***		−0.001**	−0.003***	0.18***	0.04*
CRP, mg/L	0.042***	0.033***	20.0***	17.0***	4.17***	3.33***	0.022***	0.018***	2.90***	2.28***
**PPP reagent**	**UVR**	**MR**	**UVR**	**MR**	**UVR**	**MR**	**UVR**	**MR**	**UVR**	**MR**
Age, y	0.003***	0.001**	−3.41***	−4.77***	−0.19***	−0.47***	−0.003***	−0.003***	0.27***	0.11**
Sex	0.096***	0.047***	−68.5***	−52.8***	19.06***	−19.6***	0.152***	0.129***	−10.9***	−12.8***
BMI, kg/m2	0.017***	0.005***	14.0***	12.4***	2.05***	1.24***	0.016***	0.01***	1.04***	0.34***
Smoking	0.023***	−0.021*	−16.5***		−4.53***		0.026**		−2.14***	
Waist:hip ratio	0.894***	0.132*	93.4**		28.86***	20.2*	0.583***	−0.29**	28.4***	22.6***
Total cholesterol, mg/dL	0.002***	0.003***	1.97***	1.67***	0.29***	COL	0.003***	0.004***	0.05***	−0.02*
LDL-C, mg/dL	0.003***		2.17***	COL	0.27***	COL	0.004***			
HDL-C, mg/dL	−0.005***	−0.006***			0.15***	−0.53***	−0.006***	−0.008***	0.06*	0.16***
TGL, mg/dL	0.001***		0.50***	0.30***	0.10***		0.001***		0.04***	0.05***
Blood glucose, mg/dL	0.001***	−0.001***	−0.34**	−0.96***	0.06**	−0.05*		−0.002***	0.10***	0.04*
CRP, mg/L	0.031***	0.025***	20.2***	17.1***	3.75***	3.24***	0.019***	0.015***	2.49***	2.13***

Linear regression results are presented as β-coefficients and their associated p-values. P-values were indicated as *p < 0.05, **p < 0.01, ***p < 0.001. Sex was coded as ‘'0” for men and ‘'1” for women, hence a negative β-coefficient indicates a decrease in outcome (TG) variable in women compared to men. Smoking was coded as ‘'0” for non-smokers and ‘'1” for current smokers, hence a negative β-coefficient indicates a decrease in outcome (TG) variable in smokers compared to non-smokers. Abbreviations: β-coef., β-coefficient; ETP, Endogenous thrombin potential; UVR, univariate regression analysis; MR, multiple regression analysis; COL, variable excluded from the model because of collinearity (VIF ≥ 10); BMI, Body Mass Index; LDL-C, low density lipoprotein cholesterol; HDL-C, high density lipoprotein cholesterol; CRP, C-reactive protein.

Total cholesterol levels are associated with a slightly prolonged lag time and time-to-peak, an elevated ETP and a reduced velocity index (Table 4). Triglycerides are positively associated with the lag time and time-to-peak, whereas the ETP and peak height significantly increase with increased triglyceride levels. LDL-C is posivitly associated with a prolonged lag time and time-to-peak, and an increased ETP, whereas HDL-C is associated with a reduced lag time and time-to-peak, and a lower TG peak height.

Additionally, as expected, age, sex and BMI were important influencers of TG parameters, especially, ETP, time-to-peak and velocity index. Smoking was associated with a significantly shorter lag time, and higher levels of acute phase reactant C-reactive protein were associated with a higher TG peak height and velocity index.

## Discussion

TG is a promising tool to assess blood coagulability, as it allows quantification of the global hemostatic potential of blood and/or plasma (37). Hence, TG provides information about an individual's hemorrhagic or thrombotic tendency (38–41). Over the past decades, associations between TG and the risk of bleeding or thrombosis have been reported in both prospective and case-control studies. For instance, studies have found an association of increased TG with myocardial infarction (19), coronary artery disease (42) and VTE (15, 17, 43, 44). The increased risk of cardiovascular disease (CVD)-related morbidity and mortality associated with obesity is known to reduce after weight loss (45–48). Other classical risk factors for the increased risk of CVD in individuals with high BMI are systemic inflammation (CRP), dyslipidemia and glucose intolerance (49). However, up to 20% of all coronary events occur in the absence of these risk factors, suggesting that hypercoagulability might play an important role in the relation between BMI and the occurrence of a cardiovascular event (49).

This is, to the best of our knowledge, the first large cohort study investigating the association between BMI and hypercoagulability, through TG parameters. Our findings demonstrate that overweight and obesity are associated with higher TG ETP, peak and VI. As these changes reflect hypercoagulability (29), they may thus provide an additional explanation for the increased cardiovascular risk in obese individuals. Our results are in accordance with previous studies showing that obesity is associated with alterations in the coagulation and fibrinolytic system (50). For instance, elevated levels of procoagulant factors FVII, FVIII, and fibrinogen were found in obese patients, which could explain the increased ETP (51, 52). Previously, Ay et al. found that bariatric surgery and consequent weight loss resulted in a significant reduction in TG in morbidly obese adults (22). Extrapolating these findings, it could be hypothesized that underweight (BMI <18.5 kg/m^2^) may be associated with significantly decreased TG parameters. However, in the current study population there were <0.5% (*n* = 87) underweight individuals, hence they were categorized as a “normal” BMI (i.e., up to 25).

Remarkably, the TG lag time was significantly prolonged in overweight and obese individuals compared to individuals with a normal BMI, but only for the PPP reagent low-induced TG. This is surprising, as an increased TG is often accompanied by a shortened lag time (53). The lagtime is predominantly determined by levels of tissue factor pathway inhibitor (TFPI), protein S (PS), factor VII (FVII), FIX and fibrinogen. Interestingly, a correlation between TFPI levels and BMI has been previously reported, demonstrating that an increased obesity index is associated with elevated TFPI levels (54). TFPI downregulates tissue factor-induced TG, which is reflected in a slower onset of TG, hence a prolonged lag time. The effect of TFPI is larger when PPP reagent low. Indeed, we only detected the prolonged lag time in obese patients when PPP reagent low, but not when a higher TF trigger was applied. This is in line with the reports that the PT assay, which uses a high amount of TF, does not show changes in clotting time in obese individuals (55). In addition to elevated TFPI levels in obese individuals, increased fibrinogen levels can contribute to a prolonged lag time as well. Previous reports suggest that initial traces of thrombin formed at the start of the TG measurement can bind to fibrinogen/fibrin, and thereby hinder the feedback activation of upstream coagulation factors by thrombin, and subsequently contribute to a prolongation of the lag time (56).

Regarding the blood lipid profile, we found that TG parameters were significantly higher in individuals with high total cholesterol, triglycerides and LDL-C, and low HDL-C. This is in accordance with the known effects of LDL-C and HDL-C on the risk of CVD: LDL-C is considered to be a risk factor for atherothrombosis, whereas HDL-C is reported to be protective (57), although there is controversy whether the latter applies to certain patient subgroups (58). Triglyceride levels were found to contribute significantly to both PPP reagent low and PPP reagent-induced TG parameters. It has been shown that patients with hypertriglyceridemia had higher FVII and FVIII (59) levels than patients with normal levels of triglycerides. Another study found significant correlations of total cholesterol and triglyceride levels with all procoagulant factors II, VII, IX, and X (26). Of note, in these studies, plasminogen, antithrombin levels (59), protein C and PS (26) were also significantly associated with triglyceridemia. Hence, high triglyceride levels may induce both elevated procoagulant factors and increased anticoagulant factors, suggesting a natural compensatory mechanism for the lipid-associated increase in blood coagulability. From a clinical perspective, the effect of statins in reducing the risk of CVD is known to be not only related to their capacity to normalize hyperlipidemia, but also among others to their antithrombotic effect (60). Indeed, Tripodi et al. (25) previously showed that statins reduce TG in patients with hyperlipidemia. Our data corroborate this association of hyperlipidemia with hypercoagulability, through evidence in a large population cohort.

In conclusion, our study showed significant associations of BMI and blood lipid levels with TG results. These findings aid in interpreting the results of TG in individuals with high lipid levels. Moreover, they underline the importance of hemostatic abnormalities, in addition to metabolic abnormalities, in explaining the increased risk of thrombosis in obese individuals with obesity and/or dyslipidemia. Our results indicate that there is an interplay between the altered blood lipid profile, reported alterations in coagulation factor levels, and TG, even after adjustment with the reported effect of obesity on TG (51, 52). Further studies are needed to confirm the added value of measuring TG to predict the risk of thrombosis, both in healthy individuals and subjects with obesity and dyslipidemia. As clinical follow-up data from the Moli-sani cohort are available, we are currently conducting analyses to determine the relationship between TG and clinical outcome in terms of myocardial infarction, stroke and other thrombotic events.

## Data Availability Statement

The data underlying this article will be shared on reasonable request to the corresponding author. The data are stored in an institutional repository (https://repository.neuromed.it) and access is restricted by the ethical approvals and the legislation of the European Union.

## Ethics Statement

The studies involving human participants were reviewed and approved by Catholic University of Rome, Italy. The patients/participants provided their written informed consent to participate in this study.

## Author Contributions

RdLK conceived, designed and organized the measurement of thrombin generation in the Moli-sani study, analyzed and interpreted the data, and drafted and revised the manuscript. ADiC analyzed and interpreted the data and revised the manuscript. LvdV analyzed and interpreted the data, and drafted and revised the manuscript. SC managed data collection of the Moli-sani Study and revised the manuscript. MN interpreted the data and revised the manuscript. ADeC managed the Bio-bank of the Moli-sani study. AG revised the manuscript. DH, CB, and DY participated in the data acquisition, interpreted the data, and revised the manuscript. CC participated in the data acquisition and revised the manuscript. GdG and MBD originally inspired the Moli-sani study, promoted the Authors collaboration, and revised the manuscript. BdL conceived, designed and organized the measurement of thrombin generation in the Moli-sani, study, analyzed and interpreted the data, and drafted and revised the manuscript. LI originally conceived and designed the Moli-sani study, promoted the Authors collaboration, interpreted the data, and revised the manuscript. All authors contributed to the article and approved the submitted version.

## Funding

The enrollment phase of the Moli-sani Study was conducted at the Research Laboratories of the Catholic University in Campobasso (Italy) and supported by research grants from Pfizer Foundation (Rome, Italy), the Italian Ministry of University and Research (MIUR, Rome, Italy)–Programma Triennale di Ricerca, Decreto no.1588 and IL LABORATORY, Milan, Italy. The present analyses were partially supported by the Italian Ministry of Health: Grant 2018 (PI GdG, CoPI SC, number grant: RF-2018-12367074) and Ricerca Corrente 2022-2024. The funders had no role in study design, collection, analysis, interpretation of data and in the writing of the manuscript or in the decision to submit the article for publication.

## Conflict of Interest

Synapse Research Institute is a non-for-profit organization, member of Diagnostica Stago SAS. RdLK, LvdV, MN, DH, CB, DY, and BdL are employees of Synapse Research Institute. The remaining authors declare that the research was conducted in the absence of any commercial or financial relationships that could be construed as a potential conflict of interest.

## Publisher's Note

All claims expressed in this article are solely those of the authors and do not necessarily represent those of their affiliated organizations, or those of the publisher, the editors and the reviewers. Any product that may be evaluated in this article, or claim that may be made by its manufacturer, is not guaranteed or endorsed by the publisher.

## Appendix

**Moli-sani Study Investigators**

The enrolment phase of the Moli-sani Study was conducted at the Research Laboratories of the Catholic University in Campobasso (Italy), the follow up of the Moli-sani cohort is being conducted at the Department of Epidemiology and Prevention of the IRCCS Neuromed, Pozzilli, Italy.

**Steering Committee:** Licia Iacoviello^*^°(Chairperson), Giovanni de Gaetano^*^ and Maria Benedetta Donati^*^.

**Scientific secretariat:** Marialaura Bonaccio^*^, Americo Bonanni^*^, Chiara Cerletti^*^, Simona Costanzo^*^, Amalia De Curtis^*^, Augusto Di Castelnuovo^§^, Francesco Gianfagna°^§^, Mariarosaria Persichillo^*^, Teresa Di Prospero^*^ (Secretary).

**Safety and Ethical Committee:** Jos Vermylen (Catholic Univesity, Leuven, Belgio) (Chairperson), Ignacio De Paula Carrasco (Accademia Pontificia Pro Vita, Roma, Italy), Antonio Spagnuolo (Catholic University, Roma, Italy).

**External Event adjudicating Committee:** Deodato Assanelli (Brescia, Italy), Vincenzo Centritto (Campobasso, Italy).

**Baseline and Follow-up data management:** Simona Costanzo^*^ (Coordinator), Marco Olivieri (Associazione Cuore Sano, Campobasso, Italy), Teresa Panzera^*^.

**Data Analysis:** Augusto Di Castelnuovo^§^ (Coordinator), Marialaura Bonaccio^*^, Simona Costanzo^*^, Simona Esposito^*^, Alessandro Gialluisi^*^, Francesco Gianfagna°^§^, Emilia Ruggiero^*^.

**Biobank and biochemical laboratory:** Amalia De Curtis^*^ (Coordinator), Sara Magnacca^§^.

**Genetic laboratory:** Benedetta Izzi^*^ (Coordinator), Annalisa Marotta^*^, Fabrizia Noro^*^, Roberta Parisi^*^, Alfonsina Tirozzi^*^.

**Recruitment staff:** Mariarosaria Persichillo^*^ (Coordinator), Francesca Bracone^*^, Francesca De Lucia (Associazione Cuore Sano, Campobasso, Italy), Cristiana Mignogna°, Teresa Panzera^*^, Livia Rago^*^.

**Communication and Press Office:** Americo Bonanni^*^.

**Regional Health Institutions:** Direzione Generale per la Salute - Regione Molise; Azienda Sanitaria Regionale del Molise (ASReM, Italy); Molise Dati Spa (Campobasso, Italy); Offices of vital statistics of the Molise region.

**Hospitals:** Presidi Ospedalieri ASReM: Ospedale A. Cardarelli – Campobasso, Ospedale F. Veneziale – Isernia, Ospedale San Timoteo - Termoli (CB), Ospedale Ss. Rosario - Venafro (IS), Ospedale Vietri – Larino (CB), Ospedale San Francesco Caracciolo - Agnone (IS); Casa di Cura Villa Maria - Campobasso; Ospedale Gemelli Molise - Campobasso; IRCCS Neuromed - Pozzilli (IS).

^*^Department of Epidemiology and Prevention, IRCCS Neuromed, Pozzilli, Italy

°Department of Medicine and Surgery, University of Insubria, Varese, Italy

^§^Mediterranea Cardiocentro, Napoli, Italy

Baseline Recruitment staff is available at https://www.moli-sani.org/?page_id=173

## References

[1] Global Status Report on Noncommunicable Diseases. Geneva: World Health Organization (2014).

[2] Lusis AJ. Atherosclerosis. Nature. (2000) 407:233–41. 10.1038/35025203 11001066 PMC2826222

[3] Tarchalski J Guzik P Wysocki H. Correlation between the extent of coronary atherosclerosis and lipid profile. Mol Cell Biochem. (2003) 246:25–30. 10.1007/978-1-4615-0298-2_4 12841339

[4] Law MR Wald NJ Rudnicka AR. Quantifying effect of statins on low density lipoprotein cholesterol, ischaemic heart disease, and stroke: systematic review and meta-analysis. BMJ. (2003) 326:1423. 10.1136/bmj.326.7404.1423 12829554 PMC162260

[5] Lewington S Whitlock G Clarke R Sherliker P Emberson J Halsey J. Blood cholesterol and vascular mortality by age, sex, and blood pressure: a meta-analysis of individual data from 61 prospective studies with 55,000 vascular deaths. Lancet. (2007) 370:1829–39. 10.1016/S0140-6736(07)61778-4 18061058

[6] Obesity: Preventing and Managing the Global Epidemic. Report of a WHO Consultation. World Health Organization (WHO) Technical Report Series (2000). 11234459

[7] Saydah S Bullard KM Cheng Y Ali MK Gregg EW Geiss L. Trends in cardiovascular disease risk factors by obesity level in adults in the United States, NHANES 1999-2010. Obesity. (2014) 22:1888–95. 10.1002/oby.20761 24733690 PMC4560453

[8] Hubert HB Feinleib M McNamara PM Castelli WP. Obesity as an independent risk factor for cardiovascular disease: a 26-year follow-up of participants in the Framingham Heart Study. Circulation. (1983) 67:968–77. 10.1161/01.CIR.67.5.968 6219830

[9] Stein PD Beemath A Olson RE. Obesity as a risk factor in venous thromboembolism. Am J Med. (2005) 118:978–80. 10.1016/j.amjmed.2005.03.012 16164883

[10] Vazquez LA Pazos F Berrazueta JR Fernandez-Escalante C Garcia-Unzueta MT Freijanes J. Effects of changes in body weight and insulin resistance on inflammation and endothelial function in morbid obesity after bariatric surgery. J Clin Endocrinol Metab. (2005) 90:316–22. 10.1210/jc.2003-032059 15507518

[11] Kopp CW Kopp HP Steiner S Kriwanek S Krzyzanowska K Bartok A. Weight loss reduces tissue factor in morbidly obese patients. Obes Res. (2003) 11:950–6. 10.1038/oby.2003.131 12917499

[12] Sola E Vaya A Espana F Castello R Ramon LA Hernandez-Mijares A. Plasminogen activator inhibitor-1 levels in severe and morbid obesity. effect of weight loss and influence of 4G/5G polymorphism. Thromb Res. (2008) 122:320–7. 10.1016/j.thromres.2007.10.016 18037477

[13] Ninivaggi M Apitz-Castro R Dargaud Y de Laat B Hemker HC Lindhout T. Whole-blood thrombin generation monitored with a calibrated automated thrombogram-based assay. Clin Chem. (2012) 58:1252–9. 10.1373/clinchem.2012.184077 22665918

[14] Bloemen S Huskens D Konings J Kremers RM Miszta A de Laat B. Interindividual variability and normal ranges of whole blood and plasma thrombin generation. JALM. (2017) 2:150–64. 10.1373/jalm.2017.023630 32630978

[15] Pabinger I Ay C. Biomarkers and venous thromboembolism. Arterioscler Thromb Vasc Biol. (2009) 29:332–6. 10.1161/ATVBAHA.108.182188 19228607

[16] van Veen JJ Gatt A Makris M. Thrombin generation testing in routine clinical practice: are we there yet? Br J Haematol. (2008) 142:889–903. 10.1111/j.1365-2141.2008.07267.x 18564356

[17] Besser M Baglin C Luddington R van Hylckama Vlieg A Baglin T. High rate of unprovoked recurrent venous thrombosis is associated with high thrombin-generating potential in a prospective cohort study. J Thromb Haemost. (2008) 6:1720–5. 10.1111/j.1538-7836.2008.03117.x 18680535

[18] Tchaikovski SN Rosing J. Mechanisms of estrogen-induced venous thromboembolism. Thromb Res. (2010) 126:5–11. 10.1016/j.thromres.2010.01.045 20163835

[19] Smid M Dielis AW Winkens M Spronk HM van Oerle R Hamulyak K. Thrombin generation in patients with a first acute myocardial infarction. J Thromb Haemost. (2011) 9:450–6. 10.1111/j.1538-7836.2010.04162.x 21143375

[20] Campello E Zabeo E Radu CM Spiezia L Gavasso S Fadin M. Hypercoagulability in overweight and obese subjects who are asymptomatic for thrombotic events. Thromb Haemost. (2015) 113:85–96. 10.1160/TH14-02-0156 25318550

[21] Sonnevi K Tchaikovski SN Holmstrom M Antovic JP Bremme K Rosing J. Obesity and thrombin-generation profiles in women with venous thromboembolism. Blood Coagul Fibrinolysis. (2013) 24:547–53. 10.1097/MBC.0b013e32835f93d5 23470648

[22] Ay L Kopp HP Brix JM Ay C Quehenberger P Schernthaner GH. Thrombin generation in morbid obesity: significant reduction after weight loss. J Thromb Haemost. (2010) 8:759–65. 10.1111/j.1538-7836.2010.03766.x 20102484

[23] Rota S McWilliam NA Baglin TP Byrne CD. Atherogenic lipoproteins support assembly of the prothrombinase complex and thrombin generation: modulation by oxidation and vitamin E. Blood. (1998) 91:508–15. 10.1182/blood.V91.2.508 9427704

[24] Zieseniss S Zahler S Muller I Hermetter A Engelmann B. Modified phosphatidylethanolamine as the active component of oxidized low density lipoprotein promoting platelet prothrombinase activity. J Biol Chem. (2001) 276:19828–35. 10.1074/jbc.M007506200 11278348

[25] Tripodi A Pellegatta F Chantarangkul V Grigore L Garlaschelli K Baragetti A. Statins decrease thrombin generation in patients with hypercholesterolemia. Eur J Intern Med. (2014) 25:449–51. 10.1016/j.ejim.2014.03.016 24784951

[26] Kim JA Kim JE Song SH Kim HK. Influence of blood lipids on global coagulation test results. Ann Lab Med. (2015) 35:15–21. 10.3343/alm.2015.35.1.15 25553275 PMC4272949

[27] Iacoviello L Bonanni A Costanzo S de Curtis A di Castelnuovo A Olivieri M. The Moli-sani Project, a randomized, prospective cohort study in the Molise region in Italy; design, rationale and objectives. Italian J Public Health. (2007) 4:110–8. 10.2427/5886

[28] Centritto F Iacoviello L di Giuseppe R De Curtis A Costanzo S Zito F. Dietary patterns, cardiovascular risk factors and C-reactive protein in a healthy Italian population. Nutr Metab Cardiovasc Dis. (2009) 19:697–706. 10.1016/j.numecd.2008.11.009 19303267

[29] Hemker HC Giesen P Al Dieri R Regnault V de Smedt E Wagenvoord R. Calibrated automated thrombin generation measurement in clotting plasma. Pathophysiol Haemost Thromb. (2003) 33:4–15. 10.1159/000071636 12853707

[30] Friedewald WT Levy RI Fredrickson DS. Estimation of the concentration of low-density lipoprotein cholesterol in plasma, without use of the preparative ultracentrifuge. Clin Chem. (1972) 18:499–502. 10.1093/clinchem/18.6.499 4337382

[31] World Health Organization. Waist Circumference and Waist–Hip Ratio Report of a WHO Expert Consultation. Geneva: WHO Press, World Health Organization (2011).

[32] Kyrle PA Minar E Bialonczyk C Hirschl M Weltermann A Eichinger S. The risk of recurrent venous thromboembolism in men and women. N Engl J Med. (2004) 350:2558–63. 10.1056/NEJMoa032959 15201412

[33] McRae S Tran H Schulman S Ginsberg J Kearon C. Effect of patient's sex on risk of recurrent venous thromboembolism: a meta-analysis. Lancet. (2006) 368:371–8. 10.1016/S0140-6736(06)69110-1 16876665

[34] Haidl H Cimenti C Leschnik B Zach D Muntean W. Age-dependency of thrombin generation measured by means of calibrated automated thrombography (CAT). Thromb Haemost. (2006) 95:772–5. 10.1160/TH05-10-0685 16676066

[35] Marchi R Marcos L Paradisi I. Comparison by sex between thrombin generation and fibrin network characteristics in a healthy population. Clin Chim Acta. (2015) 441:86–9. 10.1016/j.cca.2014.12.020 25542531

[36] National National Cholesterol Education Program (NCEP) Expert Panel on Detection Evaluation and and Treatment of High Blood Cholesterol in Adults (Adult Treatment Panel III). Third report of the national cholesterol education program (NCEP) expert panel on detection, evaluation, and treatment of high blood cholesterol in adults (adult treatment panel III) final report. Circulation. (2002) 106:3143–421. 10.1161/circ.106.25.3143 12485966

[37] Kim SY Kim JE Kim HK Kim I Yoon SS Park S. Influence of coagulation and anticoagulant factors on global coagulation assays in healthy adults. Am J Clin Pathol. (2013) 139:370–9. 10.1309/AJCPC5C4AGFRDKMX 23429374

[38] Dargaud Y Trzeciak MC Bordet JC Ninet J Negrier C. Use of calibrated automated thrombinography +/- thrombomodulin to recognise the prothrombotic phenotype. Thromb Haemost. (2006) 96:562–7. 10.1160/TH06-03-0179 17080211

[39] Hemker HC Giesen P AlDieri R Regnault V de Smed E Wagenvoord R. The calibrated automated thrombogram (CAT): a universal routine test for hyper- and hypocoagulability. Pathophysiol Haemost Thromb. (2002) 32:249–53. 10.1159/000073575 13679651

[40] Simioni P Castoldi E Lunghi B Tormene D Rosing J Bernardi F. An underestimated combination of opposites resulting in enhanced thrombotic tendency. Blood. (2005) 106:2363–5. 10.1182/blood-2005-04-1461 15961511

[41] Regnault V Hemker HC Wahl D Lecompte T. Phenotyping the haemostatic system by thrombography–potential for the estimation of thrombotic risk. Thromb Res. (2004) 114:539–45. 10.1016/j.thromres.2004.06.017 15507289

[42] Orbe J Zudaire M Serrano R Coma-Canella I Martinez de. Sizarrondo S, Rodriguez JA, et al. Increased thrombin generation after acute versus chronic coronary disease as assessed by the thrombin generation test. Thromb Haemost. (2008) 99:382–7. 10.1160/TH07-07-0443 18278189

[43] Brandts A van Hylckama Vlieg A Rosing J Baglin TP Rosendaal FR. The risk of venous thrombosis associated with a high endogenous thrombin potential in the absence and presence of activated protein C. J Thromb Haemost. (2007) 5:416–8. 10.1111/j.1538-7836.2007.02321.x 17116237

[44] Hron G Kollars M Binder BR Eichinger S Kyrle PA. Identification of patients at low risk for recurrent venous thromboembolism by measuring thrombin generation. JAMA. (2006) 296:397–402. 10.1001/jama.296.4.397 16868297

[45] Adams KF Schatzkin A Harris TB Kipnis V Mouw T Ballard-Barbash R. Overweight, obesity, and mortality in a large prospective cohort of persons 50 to 71 years old. N Engl J Med. (2006) 355:763–78. 10.1056/NEJMoa055643 16926275

[46] Calle EE Thun MJ Petrelli JM Rodriguez C Heath CW. Body-mass index and mortality in a prospective cohort of US adults. N Engl J Med. (1999) 341:1097–105. 10.1056/NEJM199910073411501 10511607

[47] Stevens J Cai J Pamuk ER Williamson DF Thun MJ Wood JL. The effect of age on the association between body-mass index and mortality. N Engl J Med. (1998) 338:1–7. 10.1056/NEJM199801013380101 9414324

[48] Wilson PW D'Agostino RB Sullivan L Parise H Kannel WB. Overweight and obesity as determinants of cardiovascular risk: the Framingham experience. Arch Intern Med. (2002) 162:1867–72. 10.1001/archinte.162.16.1867 12196085

[49] Khot UN Khot MB Bajzer CT Sapp SK Ohman EM Brener SJ. Prevalence of conventional risk factors in patients with coronary heart disease. JAMA. (2003) 290:898–904. 10.1001/jama.290.7.898 12928466

[50] De Pergola G Pannacciulli N. Coagulation and fibrinolysis abnormalities in obesity. J Endocrinol Invest. (2002) 25:899–904. 10.1007/BF03344054 12508953

[51] Rosito GA D'Agostino RB Massaro J Lipinska I Mittleman MA Sutherland P. Association between obesity and a prothrombotic state: the Framingham offspring study. Thromb Haemost. (2004) 91:683–9. 10.1160/TH03-01-0014 15045128

[52] Guagnano MT Romano M Falco A Nutini M Marinopiccoli M Manigrasso MR. Leptin increase is associated with markers of the hemostatic system in obese healthy women. J Thromb Haemost. (2003) 1:2330–4. 10.1046/j.1538-7836.2003.00445.x 14629465

[53] Al Dieri R de Laat B Hemker HC. Thrombin generation: what have we learned? Blood Rev. (2012) 26:197–203. 10.1016/j.blre.2012.06.001 22762893

[54] Vambergue A Rugeri L Gaveriaux V Devos P Martin A Fermon C. Factor VII, tissue factor pathway inhibitor, and monocyte tissue factor in diabetes mellitus: influence of type of diabetes, obesity index, and age. Thromb Res. (2001) 101:367–75. 10.1016/S0049-3848(00)00424-2 11297753

[55] Cvirn G Gallistl S Leschnik B Muntean W. Low tissue factor pathway inhibitor (TFPI) together with low antithrombin allows sufficient thrombin generation in neonates. J Thromb Haemost. (2003) 1:263–8. 10.1046/j.1538-7836.2003.00081.x 12871499

[56] Kremers RM Wagenvoord RJ Hemker HC. The effect of fibrin(ogen) on thrombin generation and decay. Thromb Haemost. (2014) 112:486–94. 10.1160/TH14-02-0172 24964786

[57] Barter P Gotto AM LaRosa JC Maroni J Szarek M Grundy SM. HDL cholesterol, very low levels of LDL cholesterol, and cardiovascular events. N Engl J Med. (2007) 357:1301–10. 10.1056/NEJMoa064278 17898099

[58] Annema W von Eckardstein A Kovanen PT HDL. and atherothrombotic vascular disease. Handb Exp Pharmacol. (2015) 224:369–403. 10.1007/978-3-319-09665-0_11 25522995

[59] Chan P Huang TY Shieh SM Lin TS Tsai CW. Thrombophilia in patients with hypertriglyceridemia. J Thromb Thrombolysis. (1997) 4:425–9. 10.1023/A:1008857618659 10639647

[60] Undas A Celinska-Lowenhoff M Kaczor M Musial J. New nonlipid effects of statins and their clinical relevance in cardiovascular disease. Thromb Haemost. (2004) 91:1065–77. 10.1160/TH04-02-0064 15175791

