# Supplementary material for: Increased BMI and Blood Lipids Are Associated With a Hypercoagulable State in the Moli-sani Cohort
Source: Front Cardiovasc Med. 2022 Jun 16;9:897733. doi: 10.3389/fcvm.2022.897733 (PMC9243635; doi:10.3389/fcvm.2022.897733)
Supplement: Supplementary file 1 [file Table_1.docx]

# **Supplemental data**

**Supplemental Table 1. TG parameters in different categories of BMI, stratified for sex.**

|  | Women (n=11,766) | | | | Men (n=10,780) | | | |
| --- | --- | --- | --- | --- | --- | --- | --- | --- |
| TG parameter^1^ | **BMI<25 kg/m^2^** | **BMI 25-30 kg/m^2^** | **BMI>30 kg/m^2^** | **P-value^2^** | **BMI<25 kg/m^2^** | **BMI 25-30 kg/m^2^** | **BMI>30 kg/m^2^** | **P-value^2^** |
| *PPP-Low* |  |  |  |  |  |  |  |  |
| ETP, nM*min | 1660 ± 394 | 1764 ± 408 | 1846 ± 438 | <0.001 | 1594 ± 365 | 1685 ± 397 | 1750 ± 416 | <0.001 |
| Peak, nM | 351.0 ± 86.9 | 373.6 ± 87.5 | 388.2 ± 91.9 | <0.001 | 343.6 ± 81.9 | 356.1 ± 86.9 | 367.2 ± 87.2 | <0.001 |
| Lagtime, min | 3.67 (1) | 3.95 (1) | 4.00 (1) | <0.001 | 3.92 (1) | 4.00 (1) | 4.00 (1) | <0.001 |
| TTP, min | 6.00 (2) | 6.20 (1) | 6.33 (2) | <0.001 | 6.00 (2) | 6.33 (2) | 6.33 (2) | <0.001 |
| VI, nM/min | 154.5 ± 62.0 | 167.8 ± 63.2 | 176.8 ± 67.4 | <0.001 | 154.5 ± 59.8 | 159.0 ± 62.0 | 167.1 ± 61.5 | <0.001 |
| *PPP-Norm* | |  |  |  |  |  |  |  |
| ETP, nM*min | 1673 ± 410 | 1776 ± 421 | 1858 ± 452 | <0.001 | 1607 ± 375 | 1698 ± 409 | 1764 ± 430 | <0.001 |
| Peak, nM | 359.0 ± 80.5 | 377.33 ± 81.4 | 388.7 ± 85.9 | <0.001 | 344.8 ± 75.0 | 355.1 ± 80.3 | 364.5 ± 81.1 | <0.001 |
| Lagtime, min | 2.61 (1) | 2.67 (1) | 2.67 (1) | <0.001 | 2.67 (1) | 2.67 (1) | 2.67 (1) | <0.001 |
| TTP, min | 4.9 (1) | 5.0 (1) | 5 (1) | <0.001 | 5.00 (1) | 5.18 (1) | 5.00 (1) | <0.001 |
| VI, nM/min | 159.6 ± 54.3 | 169.3 ± 56.4 | 146.0 ± 11.5 | <0.001 | 152.9 ± 52.6 | 155.5 ± 54.9 | 162.1 ± 55.4 | <0.001 |
| *PPP-Norm+TM* | |  |  |  |  |  |  |  |
| ETP, nM*min | 12.41 (9) | 12.18 (9) | 12.13 (10) | 0.649 | 12.55 (10) | 12.93 (11) | 12.15 (10) | <0.001 |
| Peak, nM | 0.81 (4) | 0.80 (4) | 0.92 (4) | 0.262 | 1.34 (5) | 1.44 (6) | 1.28 (5) | 0.007 |

*^1^ Results are presented as mean ± SD for normally distributed variables and median (IQR) for skewed variables. ^2^ P-value derived through comparisons of continuous characteristics between BMI groups using one way analysis of variance F-test for normally distributed variables and Kruskal-Wallis test for skewed variables.*

**Supplemental Table 2. General characteristics and TG parameters depending on blood lipid levels.**

| Variable^1^ | Chol<240 mg/dL | Chol≥240 mg/dL | TGL<200 mg/dL | TGL≥200 mg/dL | LDL-C<190 mg/dL | LDL-C≥190 mg/dL | HDL-C<60 mg/dL | HDL-C≥60 mg/dL |
| --- | --- | --- | --- | --- | --- | --- | --- | --- |
| n (%) | 16,824 (74.6) | 5,594 (24.8) | 19.482 (86.4) | 2.936 (13.0) | 20,917 (92.8) | 1,170 (5.2) | 13,300 (59.0) | 9,117 (40.4) |
| Age, *y* | 55.4 ± 12.1 | 56.2 ± 10.7† | 55.6 ± 12.0 | 55.7 ± 10.7^NS^ | 55.6 ± 11.9 | 56.5 ± 10.4* | 55.6 ± 11.7 | 55.6 ± 12.0^NS^ |
| Sex, *% male* | 49.2 | 43.5† | 44.4 | 70.3† | 47.5 | 42.6^#^ | 60.9 | 28.6† |
| BMI, kg/m^2^ | 28.0 ± 4.8 | 28.3 ± 4.5† | 27.8 ± 4.8 | 29.8 ± 4.4† | 28.0 ± 4.8 | 28.4 ± 4.2^#^ | 28.9 ± 4.7 | 26.8 ± 4.6† |
| Smoker, % | 22.3 | 25.2† | 21.8 | 32.1† | 22.5 | 28.7† | 26.0 | 18.8† |
| WH-ratio | 0.92 ± 0.08 | 0.93 ± 0.08† | 0.91 ± 0.08 | 0.95 ± 0.07† | 0.92 ± 0.08 | 0.94 ± 0.08† | 0.93 ± 0.07 | 0.90 ± 0.09† |
| Glucose, *mg/dL* | 95 (19) | 100 (18)† | 96 (17) | 104 (23)† | 96 (18) | 102 (17)† | 98 (20) | 95 (16)† |
| CRP, *mg/L* | 1.46 (2.15) | 1.68 (2.28)† | 1.47 (2.17) | 1.81 (2.40)† | 1.50 (2.20) | 1.73 (2.26)† | 1.68 (2.45) | 1.29 (1.86)† |
| *PPP-Low* |  |  |  |  |  |  |  |  |
| ETP, *nM min* | 1685 ± 398 | 1832 ± 431† | 1711 ± 409 | 1790 ± 421† | 1709 ± 407 | 1910 ± 431† | 1718 ± 408 | 1727 ± 416^NS^ |
| Peak, *nM* | 357.9 ± 86.4 | 382.5 ± 91.7† | 360.8 ± 87.6 | 385.3 ± 90.6† | 361.9 ± 87.6 | 390.9 ± 93.1† | 363.6 ± 89.4 | 364.6 ± 86.9^NS^ |
| Lagtime, *min* | 3.94 (1) | 4.00 (1)† | 4.00 (1) | 4.08 (1)† | 4.00 (1) | 4.33 (1)† | 4.00 (1) | 3.86 (1)† |
| TTP, *min* | 6.00 (2) | 6.33 (2)† | 6.27 (1) | 6.33 (2)† | 6.26 (1) | 6.67 (2)† | 6.33 (2) | 6.00 (2)† |
| VI, *nM/min* | 161.8 ± 62.4 | 168.6 ± 65.5† | 161.5 ± 62.6 | 176.4 ± 65.8† | 162.9 ± 62.9 | 168.8 ± 66.6^#^ | 164.2 ± 63.9 | 162.3 ± 62.2* |
| *PPP-Norm* |  |  |  |  |  |  |  |  |
| ETP, *nM min* | 1699± 411 | 1841 ± 447† | 1723 ± 422 | 1806 ± 434† | 1721 ± 419 | 1921 ± 449† | 1732 ± 421 | 1737 ± 429^NS^ |
| Peak, *nM* | 360.7 ± 80.0 | 380.7 ± 86.3† | 363.6 ± 81.3 | 379.7 ± 85.7† | 364.0 ± 81.4 | 388.2 ± 87.6† | 364.0 ± 82.8 | 368.2 ± 81.1† |
| Lagtime, *min* | 2.67 (1) | 2.67 (1)† | 2.67 (1) | 2.91 (1)† | 2.67 (1) | 3 (1)† | 2.67 (1) | 2.67 (1)† |
| TTP, *min* | 5.00 (1) | 5.00 (1)† | 5.00 (1) | 5.00 (1)† | 5.00 (1) | 5.33 (1)† | 5.00 (1) | 5.00 (1)† |
| VI, *nM/min* | 161.9 ± 55.2 | 164.8 ± 59.2^#^ | 161.8 ± 55.5 | 168.1 ± 60.5† | 162.4 ± 55.9 | 164.0 ± 60.6^NS^ | 161.8 ± 56.8 | 163.8 ± 55.4^#^ |
| *PPP-Norm+TM* |  |  |  |  |  |  |  |  |
| ETP inh, *%* | 11.98 (9) | 13.83 (11)† | 12.29 (10) | 13.31 (12)† | 12.26 (10) | 14.70 (11)† | 12.29 (10) | 12.57 (9)^NS^ |
| Peak inh, % | 0.95 (4) | 1.61 (6)† | 1.02 (5) | 1.69 (7)† | 1.05 (5) | 1.82 (6)† | 1.17 (5) | 1.03 (5)† |

*Results are presented as mean ± SD for normally distributed variables and median (IQR) for skewed variables. ^2^ P-value derived through comparisons of continuous characteristics between dichotomous lipid categories (below or above threshold) using Student’s t-test for normally distributed variables, Mann-Whitney U test for skewed variables and Pearson’s χ^2^ test for categorical variables. Significance of differences are indicated in the second column for each type of blood lipid as* ** P value<0.05, #, P value <0.01, †P value <0.001, ^NS^, not significant. Abbreviations: WH-ratio, waist-hip-ratio; PPP-Low, TG reagent with low concentration of TF; PPP-Norm (+TM), TG reagent with normal concentration of TF (+ thrombomodulin); ETP, endogenous thrombin potential; TTP, time to peak; VI, velocity index; CRP, C-reactive protein; Chol,total cholesterol; LDL-C, low-density lipoprotein cholesterol; HDL-C, high- density lipoprotein cholesterol; inh, inhibition.*

**Supplementary table 3: Determinants for TG parameters in a non-stepwise linear regression model.**

|  | Lag time | ETP | Peak | Time-to-peak | Velocity index |
| --- | --- | --- | --- | --- | --- |
| PPP reagent low | | | | | |
| Age, y | 0.002 * | -5.17 *** | -0.43 *** | -0.004 *** | 0.19 *** |
| Sex | 0.096 *** | -59.1 *** | -17.4 *** | 0.152 *** | -10.4 *** |
| BMI, kg/m2 | 0.009 *** | 12.7 *** | 1.45 *** | 0.013 *** | 0.48 *** |
| Smoking |  |  |  |  |  |
| Waist:hip ratio |  |  | 35.0 *** | -0.646 *** | 31.2 *** |
| Total cholesterol, mg/dL | 0.004 *** | 1.87 *** | 0.23 *** | 0.005 *** |  |
| LDL-C, mg/dL | COL | COL | COL | COL | COL |
| HDL-C, mg/dL | -0.009 *** |  | 0.17 ** | -0.012 *** | -0.15 *** |
| TGL, mg/dL | 0.0003 * | 0.25 *** | 0.15 *** | 0.001 * | 0.11 *** |
| Blood glucose, mg/dL | -0.001 *** | -1.02 *** |  | -0.003 *** | 0.05 * |
| CRP, mg/L | 0.033 *** | 16.8 *** | 3.31 *** | 0.018 *** | 2.25 *** |
| PPP reagent | | | | | |
| Age, y | 0.001 ** | -5.05 *** | -0.54 *** | -0.003 *** |  |
| Sex | 0.046 *** | -54.7 *** | -20.2*** | 0.133 *** | -13.2 *** |
| BMI, kg/m2 | 0.005 *** | 12.9 *** | 1.30 *** | 0.01 *** | 0.33 *** |
| Smoking |  |  |  |  |  |
| Waist:hip ratio | 0.126 * |  | 21.7 * | -0.28 ** | 22.5 *** |
| Total cholesterol, mg/dL | 0.003 *** | 1.82 *** | 0.19 *** | 0.005 *** | -0.03 ** |
| LDL-C, mg/dL | COL | COL | COL |  |  |
| HDL-C, mg/dL | -0.006 *** |  | -0.20 *** | -0.008 *** | 0.20 *** |
| TGL, mg/dL |  | 0.29 *** | 0.12 *** |  | 0.08 *** |
| Blood glucose, mg/dL | -0.001 *** | -1.03 *** | -0.05 * | -0.002 *** |  |
| CRP, mg/L | 0.025 *** | 16.8 *** | 3.12 *** | 0.015 *** | 2.10 *** |

*Linear regression results are presented as β-coefficients and their associated p-values. P-values were indicated as *p<0.05, **p<0.01, **p<0.001. Sex was coded as ‘’0’’ for men and ‘’1’’ for women, hence a negative β-coefficient indicates a decrease in outcome (TG) variable in women compared to men. Smoking was coded as ‘’0’’ for non-smokers and ‘’1’’ for current smokers, hence a negative β-coefficient indicates a decrease in outcome (TG) variable in smokers compared to non-smokers. Abbreviations: β-coef., β-coefficient; ETP, Endogenous thrombin potential; COL, variable excluded from the model because of collinearity (VIF≥10); BMI, Body Mass Index; LDL-C, low density lipoprotein cholesterol; HDL-C, high density lipoprotein cholesterol; CRP, C-reactive protein.*
